# Drug-Induced Sleep Endoscopy: Technique, Indications, Tips and Pitfalls

**DOI:** 10.3390/healthcare7030093

**Published:** 2019-07-24

**Authors:** Marina Carrasco-Llatas, Silvia Matarredona-Quiles, Andrea De Vito, Khai Beng Chong, Claudio Vicini

**Affiliations:** 1 Department of Otolaryngology, Hospital Universitario Dr. Peset., 46017 Valencia, Spain; 2 Department of Otolaryngology; Ospedale Morgagni Pierantoni, 47121 Forli, Italy; 3 Department of Otolaryngology; Tan Tock Seng Hospital, Singapore 308433, Singapore

**Keywords:** drug-induced sleep endoscopy, DISE, sedation, obstructive sleep apnea, snoring

## Abstract

Drug-induced sleep endoscopy (DISE) is a diagnostic tool to assess the upper airway of snorers and obstructive sleep apnea patients in conditions that mimic natural sleep. Although DISE appears simple and similar to awake endoscopy, there are many aspects that need to be standardized in order to obtain reliable and reproducible information. In this article, we will recommend how to reliably perform DISE, its indications, and how to obtain and interpret the information of the upper airway.

## 1. Introduction

Obstructive sleep apnea (OSA) is a common health problem that is associated with an increase in cardiovascular morbimortality, a decrease in quality of life, and a higher risk of traffic accidents due to hypersomnolence [1,2]. Standard treatment for OSA is positive upper airway pressure, usually delivered in a continuous mode, known as continuous positive airway pressure (CPAP) therapy. CPAP acts like a pneumatic splint that opens the upper airway (UA), regardless of the site of obstruction. Nevertheless, CPAP adherence can be poor, and therefore alternative therapies are necessary [3]. On the other hand, surgery has 100% compliance but has limited effect on unselected patients [4]. With the arrival of new technologies such as transoral robotic surgery (TORS) and coblation, new modalities for the treatment of tongue base obstruction is now available. The sleep surgeon needs to know the exact area or areas of upper airway collapse in order to select patients that may improve with surgery and determine the areas that need to be surgically addressed. 

Routine UA assessment is performed when patients are awake—a state with no UA collapse or oxygen desaturation. In a small percentage of OSA patients, the anatomical causes of the obstruction are obvious (e.g., grade 4 palatine and/or lingual tonsils). However, for most patients, the cause is not so obvious, as they may have long soft palate, a relatively large tongue with grade 1 tonsils, or previous tonsillectomy. Therefore, it is of utmost importance to understand the UA behavior during sleep so that tailor-made treatment can be decided for every patient. 

In the late 1970s, evaluation of the UA during natural sleep was proposed [5]. Unfortunately, observation of the UA during natural sleep is complicated and impractical in daily practice. On the other hand, with the aid of sedation, the UA can be observed dynamically to allow the sleep surgeon to have an accurate idea of what is happening during sleep and the possible treatment options. Other diagnostic tools such as MRI or CT scan under sedation are also possible. Although MRI and CT scans have the advantage of having complete visualization of the UA simultaneously, they are limited by the facilities available and the need to collaborate with the radiology department. Maneuvers or changes in the position during sleep MRI or CT scan are also almost impossible. For this reason, drug-induced sleep endoscopy (DISE) has spread all over the world and is the preferred diagnostic technique to assess the UA of patients who snore or have OSA in a state that simulates natural sleep [6]. 

## 2. Technique 

Since the first publication on DISE [7], several drugs or drug combinations have been used for sedation. Some authors also use nasal decongestion, local anesthesia, and/or anti-secretory drugs. So far, there is also no uniformity regarding how to perform the sedation. Nevertheless, this point is critical to achieve a sedation level that resembles natural sleep in order to obtain reliable DISE findings. 

In the European position paper on DISE, and its later update, Ear, Nose and throat (ENT) experts on DISE put in their collective opinion and recommended possible set-up and techniques for DISE to be reliably practiced in different centers [6,8]. 

### 2.1. Where to Perform DISE and Materials

DISE must be performed in a room where all the basic resuscitation equipment (e.g., oxygen supply) are available, because safety is of utmost importance. The room should also be quiet and dark.

In order to perform DISE, the patient should be monitored with electrocardiogram (ECG) and pulse oximeter. Monitoring of sedation depth such as Bi-spectral index (BIS) is highly recommended, as the BIS level of sedation should be between 70 and 50 when the UA is evaluated in adults [6]. Evaluation of the UA in lighter sedation may underestimate the obstruction, whereas deeper sedation may cause artificial obstruction due to over sedation [9,10,11]. Having said that, one must be aware that sedation levels observed with these devices are an estimate of actual sedation, as some people are not well sedated at those recommended levels, although the majority of adult people are. For children, these BIS levels might not be applicable. 

The thinnest possible fiber endoscope, ideally with working channel to aspirate secretions, is recommended. The authors also recommend a recording system for video and sound, if possible. The recording is useful, as the videos can be replayed to the patients to help them understand their problem better as well as the potential difficulty in addressing the obstruction. The videos may also be useful in the future if the results of the UA surgery do not achieve the desired outcomes.

### 2.2. Drugs for Sedation and Method

Based on the literature, many drugs (midazolam, propofol, dexmedetomidine, remifentanil, ketamine) are used, either alone or in combination, in order to achieve the ideal level of sedation [6]. All of them have their own advantages and disadvantages. Discussing this specific subject is beyond the scope of this article; more details are explained elsewhere [12,13]. In this manuscript, only sedation with propofol using target-controlled infusion (TCI) pump will be described, as it is the sedation method that is evidence-proven to demonstrate UA collapses resembling natural sleep. Studies comparing natural sleep and TCI-propofol sleep have shown that the critical closing pressure, UA muscle responsiveness, apnea hypopnea index (AHI), levels of sedation measured by BIS and sites of obstruction are equivalent to those observed during N2 and N3 stages of natural sleep [14,15,16,17,18,19]. It is important to note that REM sleep cannot be reproduced with propofol sedation [15]. DISE usually takes 15–30 min. Within this short duration, only N2 sleep can be achieved [20]. Nevertheless, it is during this stage that most of the events occur (unless the patient has events only in REM sleep). Therefore, DISE should be considered a snapshot of what happens during the night, but it cannot provide information for the entire night. This is a limitation of the procedure, but as-long-as there is no tool that can show us the behavior of every structure of the UA during natural sleep for the whole night, DISE will continue to be the best tool available.

It is recommended to titrate the TCI-propofol pump slowly to achieve the ideal level of sedation (BIS: 70–50, does not respond to verbal stimuli). According to Heiser et al. and our personal experience, this level of sedation is achieved when the brain concentration is approximately 3 µg/mL [9]. In order to reach this concentration as quickly as possible without causing central apneas, the pump can be programmed to 2 or 2.5 µg/mL, with an increase of 0.5 µg/mL every two minutes until the level of sedation is achieved. An addition of 1–2 mg of midazolam at the beginning of the sedation will shorten the time to the observation window, although it may also cause an increase in sneezing, making the assessment more difficult [21]. 

DISE is not a painful technique, therefore local anesthesia of the nose is not necessary. Neither is nasal decongestion [6]. Antisecretory drugs such as atropine are not recommended for routine use as atropine’s effect on the UA is unknown [6]. Nevertheless, the visualization of the UA dynamics can be extremely difficult in the presence of secretions, and therefore the physician performing DISE has to balance the pros and cons and act accordingly.

### 2.3. Maneuvers and Position

The patients are usually observed in supine decubitus position, because this is the position where most of the events occur. However, assessment of the UA in a lateral position does give additional information, especially for those patients that sleep mostly in a lateral decubitus position. If the patient’s history suggests that he or she should be assessed in a lateral decubitus position, it may be easier to start DISE in this position, including doing the necessary maneuvers, before changing to supine position. Although the initial study by the de Vries group noted that the UA findings were similar in lateral decubitus compared with supine decubitus with the head turned to one side [22], a recent study with a larger sample size proved that their initial findings were inaccurate, emphasizing the need to observe the UA in lateral decubitus [23]. 

**Figure 1:**
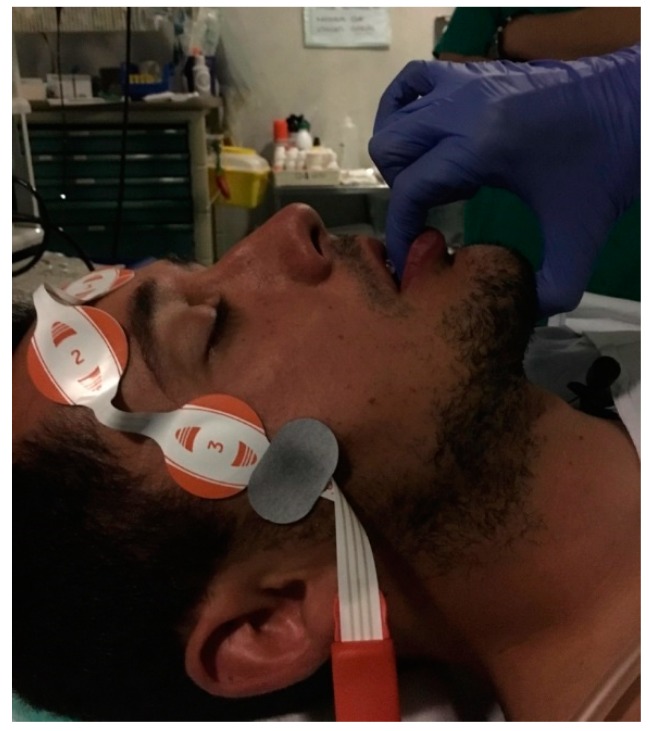
Modified Esmarch maneuverer grabbing the mandible of the patient to mimic the action of a mandibular advancement device (MAD).

It is very common for patients to open their mouths during DISE, as they usually do at home. If the mouth is open, the tongue may move backward, pushing the soft palate and further obstructing the UA. Changes to the areas and pattern of obstruction can be observed by just closing the mouth. This maneuver (often called chin-lift) is different from the Esmarch maneuver, which gently pulls the mandible forward to simulate the effect of a mandibular advancement device (MAD). As showed by the Antwerp group, maximum advancement of the mandible does not represent what a MAD can achieve [24]. Other than the advancement of the mandible, the thickness of a MAD needs to be simulated as well. Therefore, an interincisive distance of approximately 5 mm is necessary to mimic a MAD. The authors pull the mandible by grabbing it with the fingers inside the mouth in order to mimic the thickness and the protrusion of the MAD in place. This maneuver is also believed to be less painful compared with pushing the mandible at its angle (Figure 1). Performing DISE with a custom-made MAD is also feasible. DISE can begin with the MAD in place. The device can be removed after observing the UA for potential collapse for at least two cycles (stable snoring, apneas/hypopneas and breathing), and the UA can be observed for another two or more cycles with different positions and maneuvers. This is because insertion of a MAD while the patient is sedated is extremely difficult, while removal is much easier with less arousal for the patient [24,25]. 

We have previously discussed the backward movement of the tongue that pushes the soft palate causing velum collapse. The Esmarch maneuver may be useful to appreciate and differentiate the obstruction related to the tongue and soft palate. Sometimes velar collapse can contribute to secondary tongue or epiglottis collapse. It has been reported that not all UA collapses observed during DISE need to be addressed to achieve a successful result [26,27]. It is important to identify and surgically address areas of primary UA collapse, which in turn leads to improvement in areas of secondary collapse. Victores et al. proposed the use of a nasopharyngeal tube to overcome palatal collapse. After the insertion of the tube, many patients with multilevel collapse were noted to have improvement or complete resolution of the multilevel collapse. Complete resolution of tongue base collapse was rare compared to the lateral pharyngeal wall and epiglottis. This study also noted another interesting finding: the improvement of complete palatal collapse can also reduce downstream lateral pharyngeal wall and epiglottis collapse. The authors concluded that lateral pharyngeal wall and epiglottis obstruction were dependent on the complete palatal collapse. On the contrary, improving incomplete palatal obstruction did not significantly modify downstream UA morphology [28]. Although more studies should be made to confirm those results, it appears that any tongue base or epiglottis collapse observed while the palate is open should be considered primary collapse. Complete palatal obstruction can create negative pressure, leading to UA collapse downstream of the soft palate. Lateral pharyngeal wall collapse was the site most dependent on palatal collapse, followed by the epiglottis, while the tongue base appeared to be independent of the palatal obstruction. 

### 2.4. Observation Window, Events and Classification

The UA is observed once the desired sedation level is achieved, when the patient does not respond to verbal stimuli, and when a BIS signal of between 70 and 50 is reached. It is better to begin the observation at the upper part of the airway just below the choana, in order to see the palate and how it closes. Sometimes there may be tongue base or epiglottis collapse while the palate is still open. Most of the time it is impossible to see the tongue base and epiglottis, as the soft palate is closed. After observing the soft palate for 1–2 min, the scope can be advanced downward to observe the rest of the UA. After observing every level, we can proceed with the maneuvers. DISE should take approximately 15–30 min. Longer observation is not useful, as the UA will be full of saliva due to the inhibition of deglutition by propofol [20]. 

The events that must be observed are the areas of vibration and collapse as well as the shapes of the collapse. For proper assessment of the shapes of obstruction, the tip of the endoscope should be in the middle of the UA [6]. 

**Figure 2:**
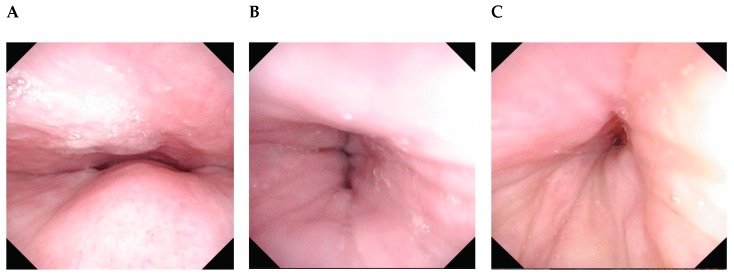

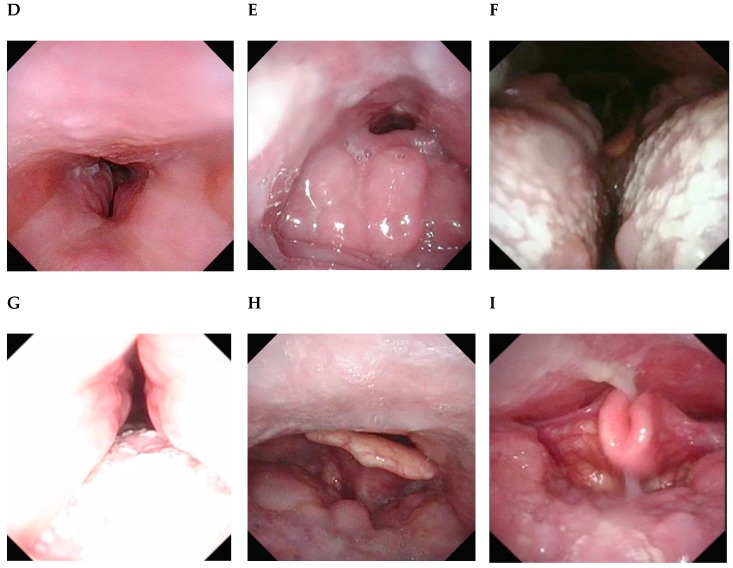
Examples of complete collapse observed at the different areas. (**A**) Velum A–P. (**B**) Velum lateral. (**C**) Velum circular. (**D**) Oropharynx lateral. (**E**) Tongue base A–P due to lymphoid hypertrophy. (**F**) Tongue base lateral, note that the tongue base bends like a book. (**G**) Tongue base concentric, there is an A–P movement of the tongue and the lateral walls also contribute to the collapse. (**H**) Epiglottis A–P. (**I**) Epiglottis lateral.

Unfortunately, there is no consensus on the ideal classification. All of them have their own advantages, but none is perfect. Sometimes it can be difficult to categorize the UA findings for the patient as the areas or shapes of collapse can change during the test. Although there is no need to use a classification system to understand the UA behavior, or to choose a tailor-made treatment for each patient, a standardized classification method is of utmost importance to compare findings between patients and results of different types of surgery. Due to its simplicity, a modified VOTE classification was recommended in the updated position paper on DISE (Table 1). Modifications include allowing any pattern of tongue base collapse and descriptive notes about the main structure causing the collapse. For example, we can indicate that the lateral wall collapse is caused by tonsillar hypertrophy, whereas tongue base collapse by lymphoid tissue. Patients with obvious lymphoid tissue hypertrophy should have a better response rate to surgery. Images of the possible complete obstructions are shown in Figure 2. 

## 3. Indications and Contraindications

DISE is a diagnostic tool that assesses the areas of vibration and obstruction of the UA. It should be performed in patients with sleep-disordered breathing, as this information is important for treatment. Common sense is essential to adjust both the patient and the media singularities. It is also important to understand that DISE does not assess the severity of the disorder. Before DISE, it is essential for the patient to have a complete polysomnography (PSG) or a simplified sleep polygraph. Some aspects of the UA anatomy of the patient are better observed while awake. Therefore, a complete awake UA examination is also important to help decide the treatment for the patient. 

As recommended by the European position paper, DISE should be performed for simple snorers and OSA patients when this information is considered important [6]. For example, it does not make sense to do DISE if the patient’s chosen treatment is CPAP. But if the patient is unable to tolerate CPAP, then DISE can help decide which treatment is best to address the problem. DISE can also be performed with the CPAP in place to understand the reason for CPAP failure. DISE is a very expensive test to be done for MAD adjustment. Nonetheless, if the patient is going for a surgery like septoplasty for deviated nasal septum, DISE can be performed in the same setting to assess the suitability of MAD as a treatment modality. At present, there are many different palato-pharyngeal surgery techniques to address palatal obstruction. Some articles suggest that various techniques may better benefit different patterns of pharyngeal collapse [29,30]. Therefore, it makes sense to perform DISE before surgery in order to help choose the appropriate technique based on the pattern of collapse.

Contraindications include patients for whom DISE cannot be safely performed, such as those with American Society of Anesthesiologists (ASA) class 4, pregnancy or with an allergy to the drugs. The last contraindication should be very uncommon, because if the patient is allergic to propofol then midazolam or dexmedetomidine can be used instead. We strongly believe that a high AHI is not a contraindication in DISE. Although the first-choice treatment for these patients is CPAP, those that cannot tolerate it still deserve an alternative treatment that may help decrease the severity of OSA. DISE will give important information in helping to choose the treatment. 

## 4. Tips and pitfalls

DISE is a very useful test, but in order to do it reliably, there are some important tips to consider:

### 4.1. Safety

If DISE is not performed in an operating room, check to make sure that the basic resuscitation equipment is available (e.g., oxygen supply, bag-valve mask).

### 4.2. Sedation

The level of sedation is crucial. The patient should not respond to verbal stimuli but still be able to respond to pain. BIS level should be between 70 and 50. The median TCI concentration of propofol is 3.2 µg/mL. 

### 4.3. Patience

The level of sedation cannot be achieved too quickly (i.e., in 2 or 3 min). If propofol is injected in bolus too quickly, the patient will be over-sedated, inducing severe UA collapse without snoring. Ideally, the UA should be observed while the patient is snoring. Although uncommon, some patients do not snore significantly. The findings of the UA should still be reliable as long as there is stable respiration at a correct BIS level. Spend more time observing the UA. 

### 4.4. Manage Secretions

If there is too much saliva it will be difficult to observe the shapes of the collapses. Aspiration of secretions with a working channel through the endoscope is the best option. If that is not possible, then careful suction with a canula through the mouth or nose can be done. We must be very careful not to injure the mucosa because proper DISE assessment cannot be done in the presence of blood. Be patient, as aspiration may also arouse the patient. 

### 4.5. Maneuvers

The Esmarch maneuver should still be performed in every patient, even when there is no tongue base collapse or when MAD is not considered a treatment option. One must remember that there are different types of tongue base [31], and the upper part of the tongue overlaps with the palate. Some palatal collapses are indeed caused by the tongue. This maneuver may help to differentiate true palatal collapse. We should also consider performing other maneuvers (e.g., lateral position and insertion of nasopharyngeal tube) as they may give additional valuable information.

### 4.6. Classification

Although there is no consensus, a classification system is useful to report the findings. It will help to compare the results between your patients and to learn from the experience of other centers.

### 4.7. Write a Report

We should report the level of sedation, lowest oxygen saturation, drug used for sedation (including the delivery mode and the dose used to achieve sedation target level), UA findings, and maneuvers performed during DISE. The updated European DISE position paper provides a useful template. 

Degree of obstruction can be: 0, no vibration obstruction less than 50%; 1, partial obstruction or vibration; or 2, complete obstruction. Use X if it is not possible to watch that area. 

## 5. Conclusions

DISE is a useful tool to watch the UA of patients with snoring or OSA in a state that mimics natural sleep if the sedation is performed carefully. These findings help to decide the best treatment for each patient when CPAP is not considered or tolerated. To obtain as much information as possible, it is important to perform maneuvers that modify the UA. Nevertheless, DISE represents a small part of the night, mostly what happens in N2, it cannot give information about REM sleep phase.

## Figures and Tables

**Table 1:** Modified VOTE classification.

**Area**	Degree of Obstruction	Configuration		
		A–P	Lateral	Circular
**Velum**				
Oropharyngeal Walls				
Tongue Base				
Epiglottis				
